# Lack of response to disgusting food in the hypothalamus and related structures in Prader Willi syndrome

**DOI:** 10.1016/j.nicl.2019.101662

**Published:** 2019-01-04

**Authors:** Laura Blanco-Hinojo, Jesus Pujol, Susanna Esteba-Castillo, Gerard Martínez-Vilavella, Olga Giménez-Palop, Elisabeth Gabau, Laia Casamitjana, Joan Deus, Ramón Novell, Assumpta Caixàs

**Affiliations:** a MRI Research Unit, Department of Radiology, Hospital del Mar, 08003 Barcelona, Spain; b Centro Investigación Biomédica en Red de Salud Mental, CIBERSAM G21, 08003 Barcelona, Spain; c Specialized Service in Mental Health and Intellectual Disability, Institut Assistència Sanitària (IAS), Parc Hospitalari Martí i Julià, 17190 Girona, Spain; d Endocrinology and Nutrition Department, Parc Taulí Hospital Universitari, Institut d'Investigació i Innovació Parc Taulí I3PT- UAB, 08208 Sabadell, Spain; e Clinical Genetics, Pediatrics Department, Parc Taulí Hospital Universitari, Institut d'Investigació i Innovació Parc Taulí I3PT- UAB, 08208 Sabadell, Spain; f Department of Clinical and Health Psychology, Autonomous University of Barcelona, 08193 Barcelona, Spain

**Keywords:** Prader Willi syndrome, Functional MRI, Disgust, Hypothalamus

## Abstract

**Objective:**

To investigate, based on a putative abnormal neural processing of disgusting signals in Prader Willi syndrome (PWS) patients, the brain response to visual representations of disgusting food in PWS using functional MRI (fMRI).

**Methods:**

Twenty-one genetically-confirmed PWS patients, 30 age- and sex-matched and 28 BMI-matched control subjects viewed a movie depicting disgusting food-related scenes interspersed with scenes of appetizing food while fMRI was acquired. Brain activation maps were compared between groups and correlated with disgust and hunger ratings.

**Results:**

At the cortical level, the response to disgusting food representations in PWS patients was qualitatively similar to that of control subjects, albeit less extensive, and engaged brain regions typically related to visually-evoked disgust, such as the anterior insula/frontal operculum, the lateral frontal cortex and visual areas. By contrast, activation was almost absent in limbic structures directly concerned with the regulation of instinctive behavior robustly activated in control subjects, such as the hypothalamus, amygdala/hippocampus and periaqueductal gray.

**Conclusions:**

Our study provides novel insights into the neural substrates of appetite control in a genetically-mediated cause of obesity. The presence of significant cortical changes further indicates that PWS patients consciously process disgusting stimuli, but the virtual absence of response in deep, limbic structures suggests that disgusting signals do not adequately reach the primary brain system for the appetite control.

## Highlights

• We report an abnormal pattern of brain response to images of disgusting food in PWS.
• The activation demonstrated by PWS patients was restricted to the cerebral cortex.
• Higher subjective disgust ratings were associated with greater insula activation.
• In contrast, the neural response was almost absent in deep subcortical structures.
• Disgusting signals may not adequately reach a main brain system for appetite control.

## 1 Introduction

Prader Willi syndrome (PWS) is a genetic disorder caused by the lack of expression of paternal genes in the chromosome 15, resulting in a complex phenotype that presents with characteristic physical traits and specific endocrine and behavioral problems (Cassidy et al., 2012). Altered control of appetite is generally a major issue for PWS patients in the form of a strong desire for food and a tendency to overeat with subsequent obesity (Cassidy et al., 2012; McAllister et al., 2011; Miller et al., 2011; Tauber et al., 2014). Although the origin of hyperphagia in PWS remains incompletely understood, it has been associated with impaired satiety mechanisms (Hinton et al., 2006; Holland et al., 1995; Shapira et al., 2005) secondary to hypothalamic dysfunction (Goldstone, 2004, Goldstone, 2006; Swaab, 1997; Swaab et al., 1995) and related structures also implicated in the regulation of feeding (Dimitropoulos and Schultz, 2008; Hinton et al., 2006; Holsen et al., 2006; Pujol et al., 2016; Zhang et al., 2013).

Although PWS patients appear to show an adequate general knowledge about food (Dykens, 2000), indiscriminate eating is a common phenomenon (Dykens, 2000; Dykens et al., 2007). Specific eating behaviors include consuming products commonly considered as unappealing or inedible, food from the trash and decayed food (Dykens et al., 2007; Russell and Oliver, 2003), which suggests the hypothesis that the altered control of appetite could partly result from the inadequate responding to disgust-evoking stimuli in brain systems regulating feeding. Besides the potential health risk of eating spoiled food (Chapman and Anderson, 2012), a deficient processing of disgusting signals could contribute to overeating and obesity in PWS via a higher threshold for rejecting food products (Houben and Havermans, 2012).

We used functional MRI (fMRI) to examine the brain response to visual representations of disgusting food in a group of PWS patients compared with a healthy control group. Participants watched a movie showing disgusting food scenes interspersed with baseline scenes of appetizing food. Brain activation maps were compared between both groups and correlated with disgust and hunger ratings.

## 2 Material and methods

### 2.1 Participants

Thirty adult PWS patients were recruited from PWS referral centers in Barcelona and Girona. Genetic testing to confirm the chromosome 15 anomaly was repeated in all patients at the time of inclusion by procedures fully described elsewhere (Pujol et al., 2016). Patients younger than 18 years, those who had nonstable medical conditions and those considered unable to follow MRI instructions were not eligible to participate in the study. Nine patients were excluded on the basis of excessive head motion during fMRI (7 cases) or insufficient imaging data collection (2 cases) resulting in a final sample of 21 patients. A total of 9 patients were taking psychiatric medication (fluoxetine, topiramate, or both, occasionally combined with antipsychotics [*n* = 4]). Treated patients were on a stable medication regime for at least 3 months prior to imaging assessment.

Thirty healthy subjects matched by age and sex to the PWS group made up the control sample. Exclusion criteria included relevant medical or neurologic disorders, substance abuse or psychiatric disease and current medical treatment or eating disorders (Pujol et al., 2018). To control for the confounding effect of obesity, we included an additional control group of thirty subjects matched by age, sex and BMI to the PWS group. Two controls were excluded from the analysis due to excessive head motion during fMRI. Table 1 provides characteristics of study participants.

**Table 1:** Demographic and clinical details of the sample.

	PWS (*n* = 21)	Normal-weight controls (*n* = 30)	Obese controls (*n* = 28)
Age, yr	27.8 ± 8.3 (18–47)	27.9 ± 7.8 (19–45)	28.8 ± 7.2 (19–46)
Sex, M/F	12/9	15/15	15/13
Body mass index, kg/m^2^	30.5 ± 6.3 (21.5–43.4)	22.1 ± 2.0 (17.7–25)	33.1 ± 5.7 (26.1–44.4)
IQ K-BIT - Total score	68.7 ± 11.4 (48–92)	111.0 ± 11.2 (90–131)^a^	110.9 ± 10.4 (91–137)^b^
Verbal	76.3 ± 11.8 (50–99)	111.3 ± 12.3 (89–133)	110.9 ± 11.9 (84–133)
Performance	72.7 ± 13.2 (44–102)	112.4 ± 8.4 (93–127)	112.2 ± 10.3 (89–136)
Genetic subtype, no.			
Type I deletion	5		
Type II deletion	8		
Uniparental disomy	5		
Imprinting defect	3		
Hyperphagia Questionnaire^c^			
Total score (range 0–44)	18.6 ± 9.5 (4–38)	0 ± 0 (0–0)	1.9 ± 1.7 (0–7)
Behavior (range 0–20)	9.1 ± 5.2 (2−20)	0 ± 0 (0–0)	0.6 ± 1.0 (0–4)
Drive (range 0–16)	6.9 ± 3.7 (1–14)	0 ± 0 (0–0)	0.8 ± 1.1 (0–3)
Severity (range 0–8)	2.6 ± 2.7 (0–8)	0 ± 0 (0–0)	0.5 ± 0.9 (0–3)

Values are expressed as group: mean ± standard deviation (range). PWS, Prader Willi syndrome; IQ - KBIT, Intelligence Quotient - Kaufman Brief Intelligence Test.

a n = 28.

b n = 27.

c Dykens et al., 2007.

The study was approved by the Clinical Research Ethical Committee-Corporació Sanitària Parc Taulí of Sabadell, Barcelona, and conducted according to the principles expressed in the Declaration of Helsinki. Written informed consent was obtained from caregivers and control subjects. Verbal or written assent was also obtained from the PWS patients.

### 2.2 Stimulus and experimental procedure

A 6-m color movie was used involving the successive presentation of appetizing food-related scenes as baseline condition and 8 scenes of disgusting food interspersed throughout the movie at pseudorandom intervals (Pujol et al., 2018). Each disgusting scene lasted 8–10 s. In the appetizing condition, the scenes presented palatable food items in restaurants and gastronomy scenes, whereas the disgust-evoking scenes showed food-related disgust elicitors such as decaying food, maggots and cockroaches in food and people eating live worms.

Participants underwent fMRI approximately 60 min after receiving a standardized meal to maximally control for the experimental context. During image acquisition participants were instructed to lay still and passively view the movie displayed using MRI-compatible high resolution goggles (VisuaStim Digital System, Resonance Technology Inc., Northridge, CA, USA). Immediately after scanning, participants were asked to rate the intensity (on a 0–100 scale) of the elicited feeling of disgust experienced during the fMRI experiment. Participants also indicated their subjective feeling of hunger immediately before entering the MRI room.

### 2.3 MRI acquisition

Brain images were acquired on a 1.5-T Signa Excite system (General Electric, Milwaukee, WI, USA) with an eight-channel phased-array head coil and single-shot echo planar imaging (EPI) software. Functional images were acquired with gradient recalled acquisition in the steady state (time of repetition [TR] = 2000 ms, time of echo [TE] = 50 ms, pulse angle 90°, field of view 24 cm, 64 × 64 matrix, slice thickness of 4 mm, 1.5 mm gap). Twenty-two interleaved slices were prescribed parallel to the anterior-posterior commissure line covering the whole brain. A 6-min scan was acquired for each participant, generating 180 whole brain EPI volumes.

### 2.4 Image preprocessing

Imaging data were preprocessed and analyzed using the Statistical Parametric Mapping software package (v8) implemented in Matlab (The MathWorks Inc., Natick, MA). Functional images were realigned (motion corrected) to the first volume using conventional procedures, spatially normalized to the standard SPM-EPI template and resliced to 2-mm isotropic resolution in Montreal Neurological Institute (MNI) space. Smoothing was executed with an 8 mm full-width at half-maximum (FWHM) Gaussian filter. All image sequences were inspected for potential acquisition and normalization artifacts.

### 2.5 Control of potential head motion effects

The time series were aligned to the first image volume in each participant. Motion-related regressors and estimates of global brain signal fluctuations were included as confounding variables in our single-subject analyses. Within-subject, censoring-based MRI signal artifact removal (scrubbing) (Power et al., 2014) was used to discard motion-affected volumes. For each participant, a motion summary measurement that combined translations and rotations was computed in mm (Pujol et al., 2014). Average inter-frame motion measurements (head position variations of each brain volume as compared to the previous volume) served as an index of data quality to flag volumes of suspect quality across the fMRI run. We excluded participants showing >45 (25%) image volumes with inter-frame motion >0.2 mm. Using this criterion, a mean of 15.9 (8.8%) volumes from the total volumes that are included in the fMRI sequence were removed in patients, and a mean of 2.7 (1.5%) volumes and 11.4 (6.3%) volumes were removed in healthy and BMI-matched controls, respectively. Remaining potential motion effects were controlled by including a motion summary measurement for each participant as a covariate in the group analyses (Pujol et al., 2014). Lastly, a high-pass filter was used to remove low-frequency noise (cut off period = 1/128 Hz).

### 2.6 fMRI data analysis

In the single-subject (first) level analysis, an SPM contrast map for the main effect of task (disgust vs. appetizing condition) was generated for each participant. For this analysis, the response at each voxel was modeled using a boxcar regressor for disgust and motion-related regressors as confounding variables. The regressor of interest was first generated considering an implicit baseline condition including the appetizing scenes and an activation condition including the disgusting scenes, and was then adjusted using results of a previous group analysis of an independent sample with the same experimental setup (Pujol et al., 2018). Briefly, using a ROI based approach and MarsBar tools (Brett et al., 2002), the functional MRI signal time course from 4 preselected regions-of-interest showing the most consistent activation in previous research (frontal cortex, insula, amygdala and fusiform gyrus [Kirby and Robinson, 2017]) were averaged in each of 15 healthy participants (7 males, mean ± SD age: 26.6 ± 6.2 years) to obtain a single activation time course representing activation in core (disgust) regions. Results showed that, on average across the 8 scenes, disgust stimulation activated these regions with a 3 s delay and the total mean duration of core region activation was 15 s (Pujol et al., 2018).This dynamic information served to modify our baseline-activation regressor to adjust both activation onset and response duration to our 8 disgusting events. The dynamically adjusted regressor served then to carry out the individual (first-level) analyses in our study sample. The experimental paradigm is shown in Supplementary Fig. 1.

Resulting first-level SPM contrast images for each subject were carried forward to (second) group-level non-parametric permutation-based analyses using the Statistic nonParametric Mapping (SnPM13; http://warwick.ac.uk/snpm) toolbox available as an extension of the SPM package. Non-parametric interrogation was selected for examination of second-level statistical inference given the conservative nature and general robustness of the procedure with relatively small sample sizes (Nichols and Holmes, 2002). One-sample and two-sample *t*-test designs were used to estimate significant within- and between-group activation effects. In addition, SnPM voxel-wise linear regression analyses were performed in the PWS group to map the association between self-report ratings of disgust and hunger (separately) and activation across the whole brain. The Kbit total score was included as a covariate in the correlation analyses as there was a trend for patients with lower IQ to report higher disgust ratings (*r* = −0.44, *p* = .064). Data from a region showing significant correlation with self-report ratings was extracted to generate a plot illustrating the results.

#### 2.6.1 Temporal analysis of the brain response to disgust

To illustrate the temporal evolution of the brain response to visual representations of disgusting food in PWS patients, an analysis of the activation dynamics was conducted using procedures fully described in a previous study (López-Solà et al., 2010). Briefly, the finite impulse response (FIR) analysis approach (Dale and Buckner, 1997) was employed to obtain 12 activation maps, with a temporal resolution of 2 s (1 scan), covering the activation cycle and starting from the first scan after stimulus onset (time 0) to a total of 24 s (12 consecutive scans). The model involved a total implicit baseline of 176 s (88 scans). For each subject, contrast images were calculated for the 12 regressors that expressed the relative BOLD signal change from baseline throughout the activation cycle. The contrast images were then entered in 12 group analyses (one-sample *t*-tests) to generate whole-brain activation t-statistic maps for each scan. To graphically represent the group's time series response for the main significant brain areas, we plotted activation measurements (t-values obtained from the region coordinate showing peak activation across the cycle) against the 12 time points (scans).

#### 2.6.2 Thresholding criteria

To correct our results for multiple comparisons, non-parametric permutation testing was conducted using standard SnPM procedures. For all statistical models employed, we used cluster-wise inference with family-wise error (FWE) rate correction of P_FWE_ < 0.05. Input parameters to SnPM included a cluster-forming threshold of *p* < .005 and 10,000 random permutations. No variance smoothing was carried out, with the exception of the correlation models (FWHM 8x8x8) because of the low degrees of freedom in this analysis

## 3 Results

### 3.1 Subjective ratings

Prior to fMRI acquisition, PWS patients reported stronger feelings of hunger than control participants (mean ± SD, patients 39.5 ± 39.4, controls 6.5 ± 12.9; *t* = 3.6 *p* = .002), despite the fact that ratings were obtained after a standard meal in both groups.

Self-report disgust ratings in PWS patients indicated that disgusting scenes during the fMRI experiment overall evoked moderate-to-high subjective disgust at a level similar to that reported by control subjects (group mean ± SD score in patients, 68.6 ± 33.3; controls 59.4 ± 28.3, between-group differences, *t* = 1.0, *p* = .32).

### 3.2 Brain response to disgust

**Fig. 1:**
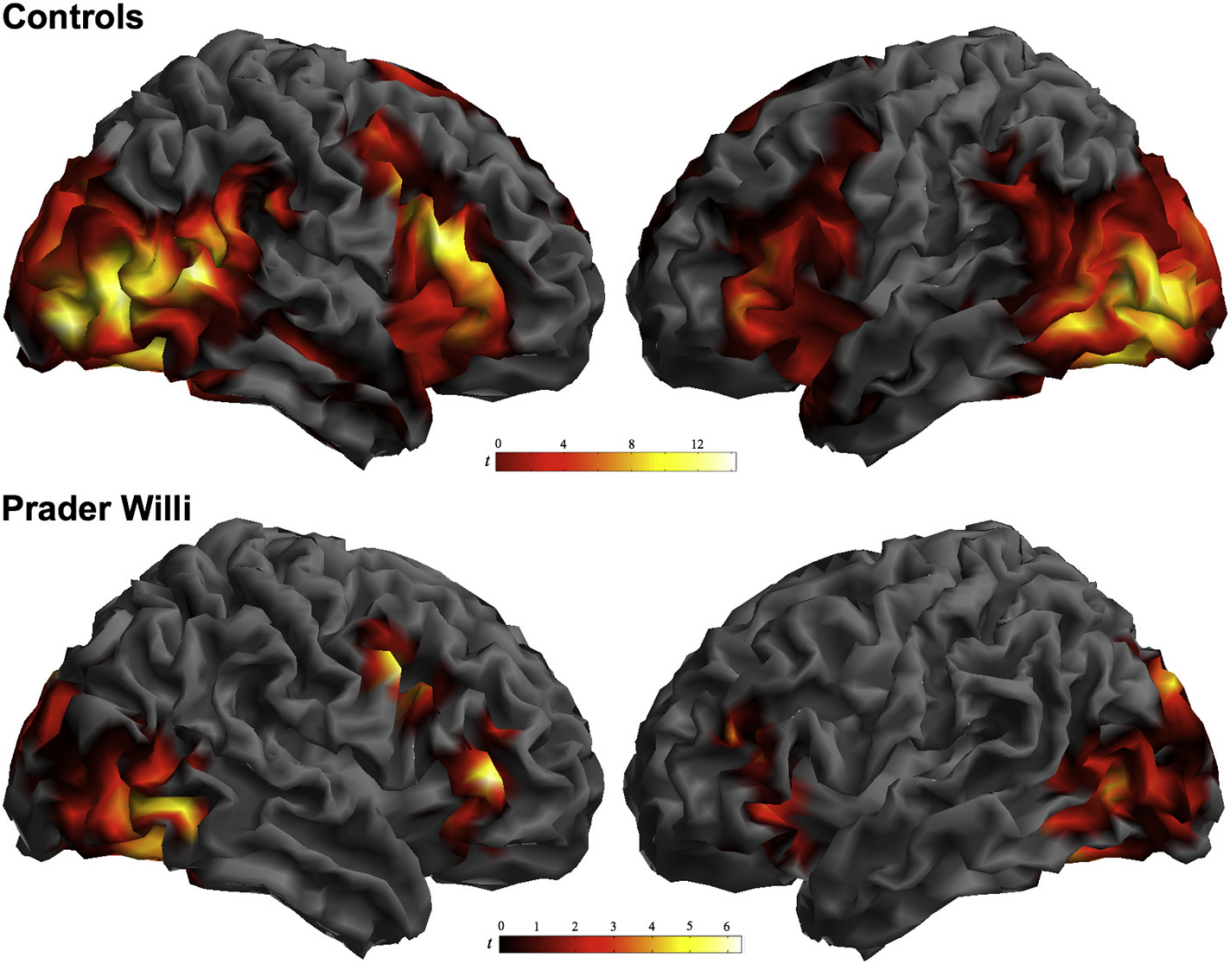
Global brain response to disgusting food scenes compared with scenes of appetizing food. The analysis was adjusted to the actual brain response of core regions characterized using dynamic information from an independent experiment (see section 2.Methods).

**Fig. 2:**
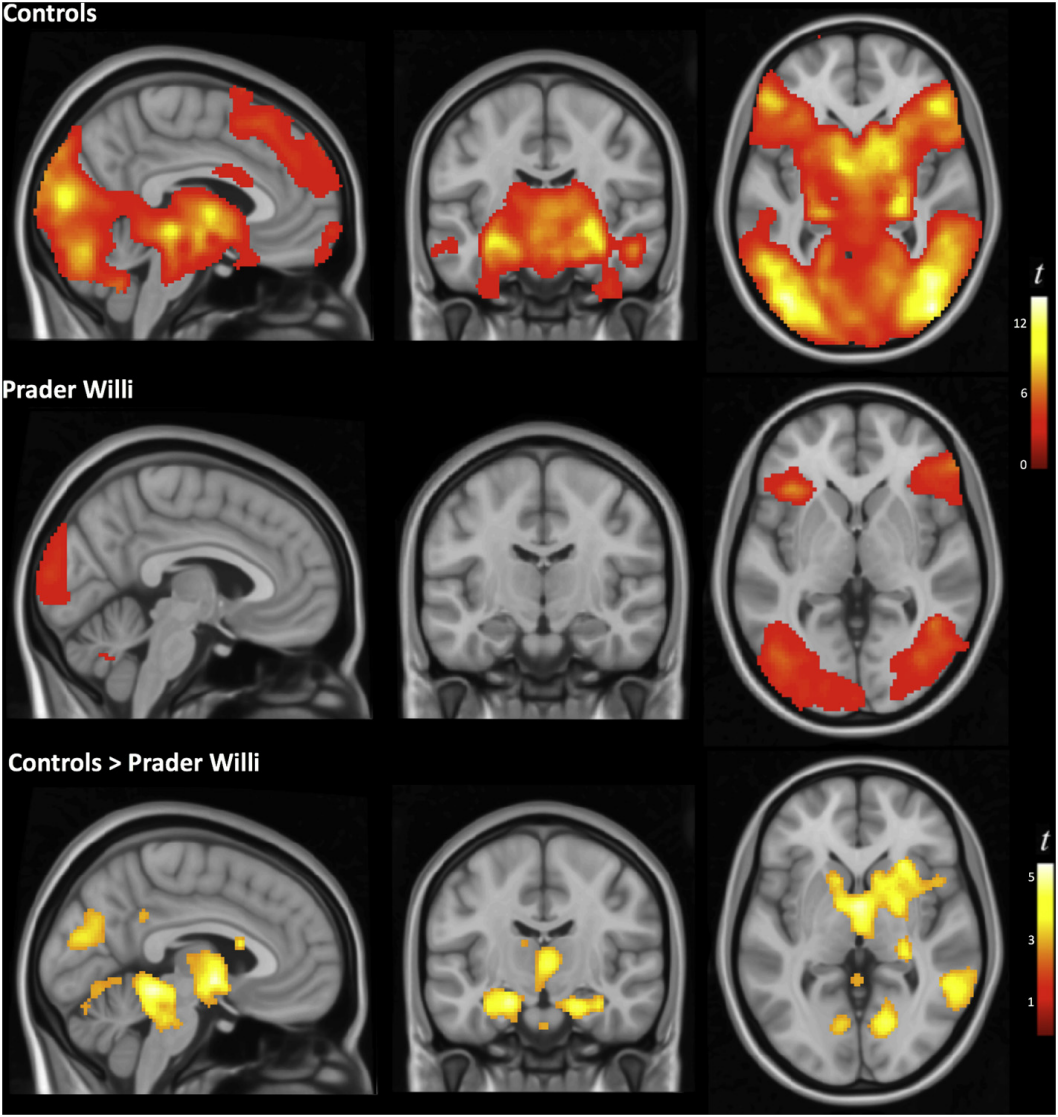
Between-group differences in the brain response to disgusting food representations. Compared with control subjects, individuals with Prader Willi syndrome showed significantly decreased activation in several subcortical structures. Right side of the figure corresponds to the right hemisphere for both coronal and axial views.

When presented with scenes of disgust (vs. appetizing food), adults with Prader Willi syndrome showed bilateral activation in distributed cortical areas encompassing the frontal operculum extending toward the anterior insula, the lateral frontal cortex, medial and lateral occipital areas and the fusiform gyrus (Fig. 1, Fig. 2). At the cortical level, the pattern of activation of the PWS group was qualitatively similar to that of the control group, albeit less extensive. Significant between-group differences were found in the right superior temporal/supramarginal gyrus, medial occipital areas and the cerebellum (Supplementary Table 1, Fig. 1, Fig. 2). At the subcortical level, however, the presentation of disgust-related images in the PWS group did not led to any significant activation of regions that were seen robustly activated in control subjects. Specifically, significantly lower activation than in the control group was observed mostly bilaterally in a cluster encompassing the amygdala, hippocampus and parahippocampal region, basal ganglia involving the ventral striatum, thalamus, hypothalamus and periaqueductal gray (PAG) matter (Supplementary Table 1 and Fig. 2). Conversely, patients did not demonstrate increased activation than controls in any region.

Similar results were observed in the comparison with the BMI-matched control group (Supplementary Figs. 2 and 3 and Supplementary Table 2). Overall, the pattern of activation of the BMI-matched control group was comparable to that of normal-weight control subjects.

**Fig. 3:**
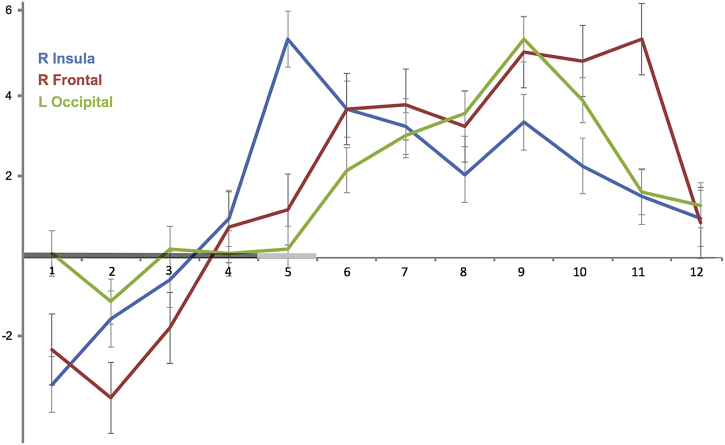
Graphic illustration of the temporal evolution of the response to disgusting food representations identified in Prader Willi syndrome patients. Statistic t values of the activations (axis *y*) are plotted against time (axis *x*) expressed as 2-s frames starting from stimulus onset (frame 1). The bold line in axis *x* indicates stimulus duration (8–10 s) of the disgusting scenes. Data from the 8 scenes were averaged to illustrate the activation cycle. The activation was measured using the t-values of the region coordinate in the cluster showing peak activation across the cycle (Supplementary Table 3).The error bars represent the standard error of the mean (SEM).

Fig. 3 illustrates the temporal evolution of the cortical response to disgusting food representations identified in PWS patients. Frame by frame measurements in representative regions covering the activation cycle (average across the eight scenes) revealed a gradual engagement of localized cortical areas reaching a peak in the insula in the fifth frame of the fMRI acquisition (the interval 8–10s after stimulus onset) and persisting broadly until the end of our temporal window (i.e., for a total of 24 s) (Supplementary Table 3).

### 3.3 Correlations between subjective scores and brain activation

**Fig. 4:**
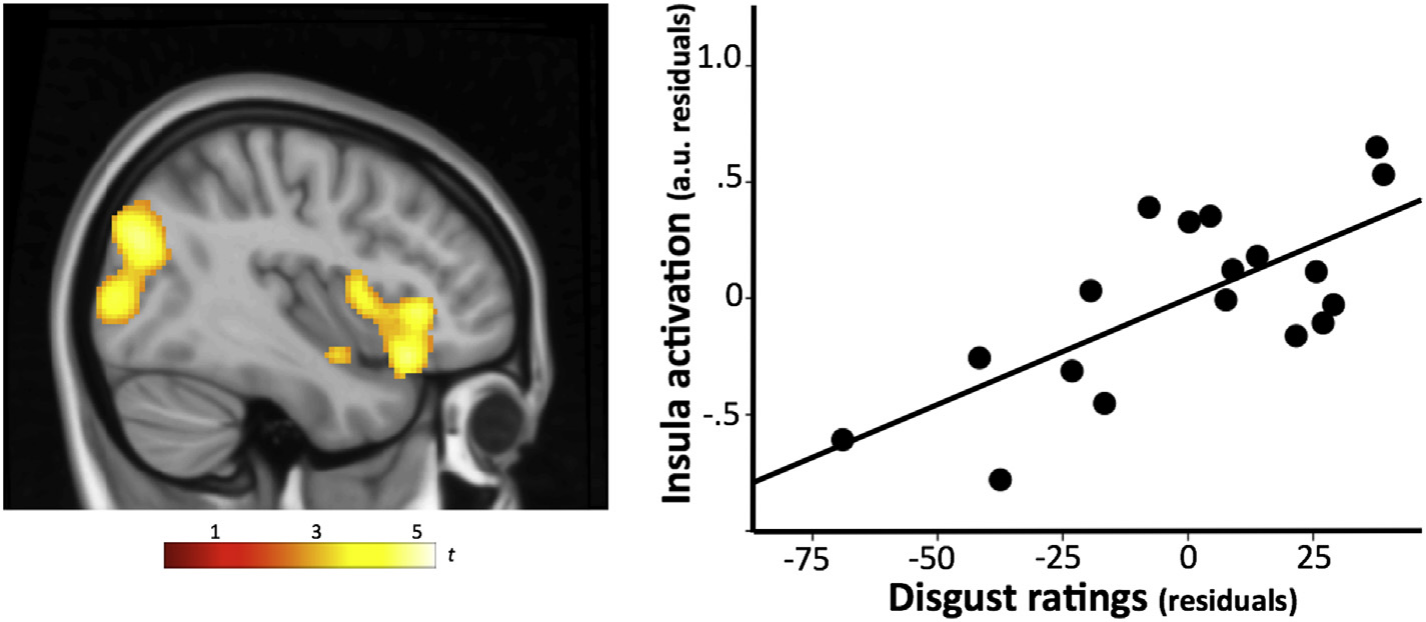
SnPM map of the correlation between subjective disgust ratings and brain activation in response to the disgusting food scenes (left panel).The scatter plot (right panel) illustrates the partial correlation (controlling for IQ) between self-report disgust ratings and activation in the right anterior insula during scenes evoking disgust (*r* = 0.71, *p* = .001). Values on the x- and y-axis correspond to residuals. Color bar represents pseudo-t values. The sagittal view shows the right hemisphere.

Within the PWS group, SnPM whole-brain analyses revealed a significant positive correlation between subjective disgust ratings during fMRI and brain activation in two clusters centered around the anterior insula (pseudo *t* = 5.4, p_FWE_ = 0.02; cluster peak, x = 44, y = 24, z = −16, *p* = .0001) and extrastriate visual cortex (pseudo *t* = 4.5, p_FWE_ = 0.02; peak, x = 40, y = −76, z = 28, *p* = .0008). Fig. 4 shows the correlation map and scatter plot illustrating these associations. No significant association was found between subjective hunger ratings and brain activation.

## 4 Discussion

The results of the present study provide evidence that individuals with PWS display an abnormal pattern of neural activity in response to disgusting food visual representations. Whilst the brain response in healthy subjects sequentially engaged a variety of functional networks involving cortical and subcortical elements (Pujol et al., 2018), the pattern of activation demonstrated by PWS patients was restricted to the cerebral cortex. Specifically, viewing disgusting scenes in the PWS group was followed by bilateral activation in the frontal opercular-insular region, lateral frontal cortex and visual areas. However, the neural response was almost absent in deep structures of the limbic brain robustly activated in control subjects, such as the hypothalamus, amygdala/hippocampus, and PAG.

The anterior portion of the insula and frontal operculum are considered to contain the primary gustatory cortex (Scott and Plata-Salamán, 1999) and receive somatosensory (oral cavity) and visceral (gut) inputs required for taste representation (Dagher, 2012). Imaging studies indicate that neural activity in these areas responds to physical gustatory stimuli but also to the sight of disgusting foods (Calder et al., 2007) and the view of facial expressions of disgust (Krolak-Salmon et al., 2003; Phillips et al., 1997). In PWS patients, the insula and frontal operculum were activated and a significant correlation was found such that higher subjective disgust ratings were associated with increased activation in the right anterior insula. These were the most consistent findings observed in PWS patients and suggest that commonly disgusting stimuli may generate conscious sensations in the distaste/disgust domain.

The lateral-ventral frontal cortex, also significantly activated in PWS patients, constitutes a main element of the frontoparietal network involved in consciously re-orienting attention to salient sensory stimuli (Corbetta et al., 2008; Downar et al., 2001). Behaviorally relevant visual features, as may be those related to the quality of food, elicit activation in this network (Corbetta et al., 2008; Frank and Sabatinelli, 2012). The brain response in subjects with PWS included the frontal element of this functional network, suggesting some implicit capability of patients to process disgusting scenes as salient stimuli in the background of appetizing food. However, in contrast to reference control subjects, patients demonstrated poor activation in the right temporo-parietal junction, the posterior element of the salience network (Corbetta and Shulman, 2002), which suggests that the stimuli salience processing may be incomplete.

Activations in primary and secondary visual regions are part of the normal response to emotion-evoking visual stimuli (Phan et al., 2002; Stark et al., 2007). The lateral occipital and fusiform areas appear to be particularly sensitive to disgusting stimuli (Karama et al., 2011). A recent imaging study in healthy adults showed that the processing of inedible food activate visual-associative brain regions to a greater degree than edible food (Becker et al., 2016). Significant visual cortex activation in our study further suggests that PWS patients distinguish edible from inedible visual representations of food. However, the early component of the visual activation, occurring in healthy subjects with a short delay of 2 s (Pujol et al., 2018), was not present in patients. The initial visual response to salient stimuli is thought to be modulated and reinforced by deep structures including the amygdala and the PAG (Bradley et al., 2003). No deep brain activation was observed in patients, which is in the same direction as the lack of the early component of the visual activation.

The processing of disgusting stimuli in healthy subjects also involves robust activation of a number of interconnected primitive/limbic brain structures commonly implicated in the regulation of instinctive behavior (Pujol et al., 2018). They include the PAG (a pivotal element in the mediation of automatic responses to potential danger), the amygdala/hippocampus (ingredients common to all basic emotions that are responsible for threat context learning) and the hypothalamus (the main center for regulating food intake) (Pujol et al., 2018). Activation was virtually absent in all of these structures in PWS patients in our study. Therefore, it appears that PWS patients are to some extent capable of processing disgusting stimuli at the cortical, conscious level, but their limbic brain systems are notably insensitive to this sort of stimulation.

The lack of limbic brain response may be relevant to the extent it could explain altered eating behavior and subsequent obesity in PWS, which is considered to be a failure of satiety (McAllister et al., 2011; Tauber et al., 2014) potentially related to hypothalamic dysfunction (Goldstone, 2006). The hypothalamus and the amygdala are strongly connected to each other and both to the basal ganglia/orbitofrontal loops that process satiety signals. We have recently reported abnormal functional connectivity between basal ganglia/orbitofrontal loops and both the hypothalamus and the amygdala associated with obsessive eating behavior in PWS patients (Pujol et al., 2016). Although the exploration of anatomical data was outside the scope of this study, it is worth mentioning here that volumetric results from an ongoing investigation by our group examining praxis skills in the same subjects (in preparation), show a pattern of extensive gray matter reduction in PWS patients involving predominantly the cerebral cortex. However, the subcortical structures showing low response to disgusting food were notably preserved with little overlap between functional and anatomic alterations (Supplementary Fig. 4), which emphasizes the relative specificity of the current findings.

We would succinctly mention the commonality of our findings with other neurodegenerative disorders. At the extreme of disgust-related disorders, coprophagia is associated with a range of neuropsychiatric disorders, especially in demented patients with evidence of medial temporal lobe and amygdala atrophy (Josephs et al., 2016). Damage at both locations is in fact the neuroanatomical substrate of the Klüver-Bucy syndrome that is characterized by hyperorality in association with other compulsive symptoms and generally diminished fear responses (Lanska, 2018). However, in the behavioral variant of frontotemporal dementia, abnormal eating behavior has also been related to hypothalamic degeneration, with potential involvement of its connections with other structures controlling eating (Ahmed et al., 2015; Bocchetta et al., 2015). The deficient response to disgust in our PWS patients involved both amygdala and hypothalamus, which showed no evident tissue atrophy. Other data indicate that the origin of disgust-related symptoms may be notably subtle. For example, pica, as the compulsive eating of non-nutritive substances, is a reversible phenomenon associated with iron deficiency, relatively frequent in pregnant women and preadolescents (Borgna-Pignatti and Zanella, 2016).

Noteworthy, the absence of limbic response in the PWS patients occurred even when self-report ratings of disgust did not differ between groups. This observed dissociation between brain response and subjective ratings in PWS is consistent with family reports and data from behavioral studies in PWS highlighting a bias in their eating behavior such that despite showing an adequate general knowledge about food (i.e., what is and isn't considered acceptable to eat), they do not care to eat oddly combined and visibly contaminated food items (Dykens, 2000). It is possible then, that they know that they mustn't eat certain food items and adhere to socially acceptable responses (e.g., spoiled food is disgusting), in spite of their indiscriminate eating behavior at the instinctive level.

The IQ was used as a confounder in the correlation analyses as there was a trend for patients with lower IQ to report higher disgust ratings. Adding this variable, however, did not change the pattern of results. Other cognitive and behavioral measures used in our previous study on obsessive-compulsive phenomena in PWS (Pujol et al., 2016) particularly related to the inhibitory control of behavior, such as the presence and severity of compulsions, self-picking and hyperphagic symptoms, were also considered here in relation to disgust, but we did not find any significant association. By contrast, in the previous study we did observe a correlation between inhibitory control alterations in patients (e.g. skin picking, obsessive eating behavior) and abnormal functional connectivity in pathways connecting frontal to subcortical structures involved in different aspects of executive control. Future imaging studies may help to address whether other clinical expressions of the assorted PWS are associated with particular brain activation patterns.

The participants' internal state, particularly regarding hunger levels, may also influence the motivational value of the food representations (i.e., automatic tendencies of withdrawal) and thus modulate the feelings of disgust. As revealed by previous studies investigating the specific influence of homeostatic dysregulation on disgust, hungry subjects exhibit weaker disgust reaction toward unpalatable foods than satiated participants (Hoefling et al., 2009). In our study, all participants received a standardized meal an hour prior to fMRI. Although hunger ratings immediately before the scanning were significantly higher in PWS patients, group mean scores were low in both cases and no significant association was found between the evoked brain activation in PWS and self-report ratings of hunger.

Some limitations of this study should be considered. Because our visual stimulus featured food-related dynamic scenes continuously throughout the experiment, we cannot infer from our results that subcortical areas showing lack of significant activation in PWS (e.g., amygdala, hypothalamus) are always inactive, but only that they are not more active during disgusting than during appetizing food scenes. Based on results of previous studies in PWS showing a strong emotional response to food images relative to control subjects (Hinton et al., 2006; Miller et al., 2007; Key and Dykens, 2008), it is likely that the limbic system is insensitive to the disgusting attributes of food, but no to food representations themselves. Future studies using an additional baseline condition (e.g. neutral stimuli) may help elucidating this question. In addition, a limitation when assessing patients with a degree of intellectual disability using fMRI is the risk of excessive head motion that may reduce activation detection sensitivity. We have stressed on this point adopting several procedures in order to control for the effect of motion, including a rigorous exclusion of individuals with less optimal image quality.

### 4.1 Conclusion

Our study provides novel insights into the potential origin of eating behavior alterations and subsequent obesity in patients with PWS by characterizing the brain response to disgusting food representations using fMRI. The presence of significant cortical changes further indicates that PWS patients to some extent consciously process disgusting stimuli. This is important to the extent that it suggests that perception and the understanding of disgust are not the major handicaps in PWS patients. Results from the behavior testing were clearly in that direction, showing that PWS patients identified disgusting food to the level of control subjects. Their eating control problems seem to be originated at a more basic/instinctive level of appetite control, as the virtual absence of response in the hypothalamus and related structures suggests. These results may support the potential feasibility of therapeutic interventions for PWS aimed at promoting learned aversive responses (e.g., teaching appropriate aversive reactions to the idea of eating spoiled food from the garbage) in the absence of a strong negative or disgust instinctive reaction.

## Appendix A Supplementary data

Supplementary material Image 1
