# Supplementary material for: Lack of response to disgusting food in the hypothalamus and related structures in Prader Willi syndrome
Source: Neuroimage Clin. 2019 Jan 4;21:101662. doi: 10.1016/j.nicl.2019.101662 (PMC6412080; doi:10.1016/j.nicl.2019.101662)
Supplement: Supplementary file 1 — Supplementary material [file mmc1.pdf]

Lack of response to disgusting food in the hypothalamus and related structures in Prader Willi syndrome

(Blanco-Hinojo et al.)

Supplementary Material

Supplementary Figure 1. Experimental paradigm.

Supplementary Figure 2. Global brain response to disgusting food scenes in PWS patients and BMI-matched controls.

Supplementary Figure 3. Differences in brain activation between PWS patients and BMI-matched controls.

Supplementary Figure 4. Combined representation of functional and volumetric results in PWS patients.

Supplementary Table 1. Regions showing significant activation differences between PWS patients and normal-weight controls.

Supplementary Table 2. Regions showing significant activation differences between PWS patients and BMI-matched controls.

Supplementary Table 3. Peak activations observed during the disgust-evocation cycle in PWS patients.

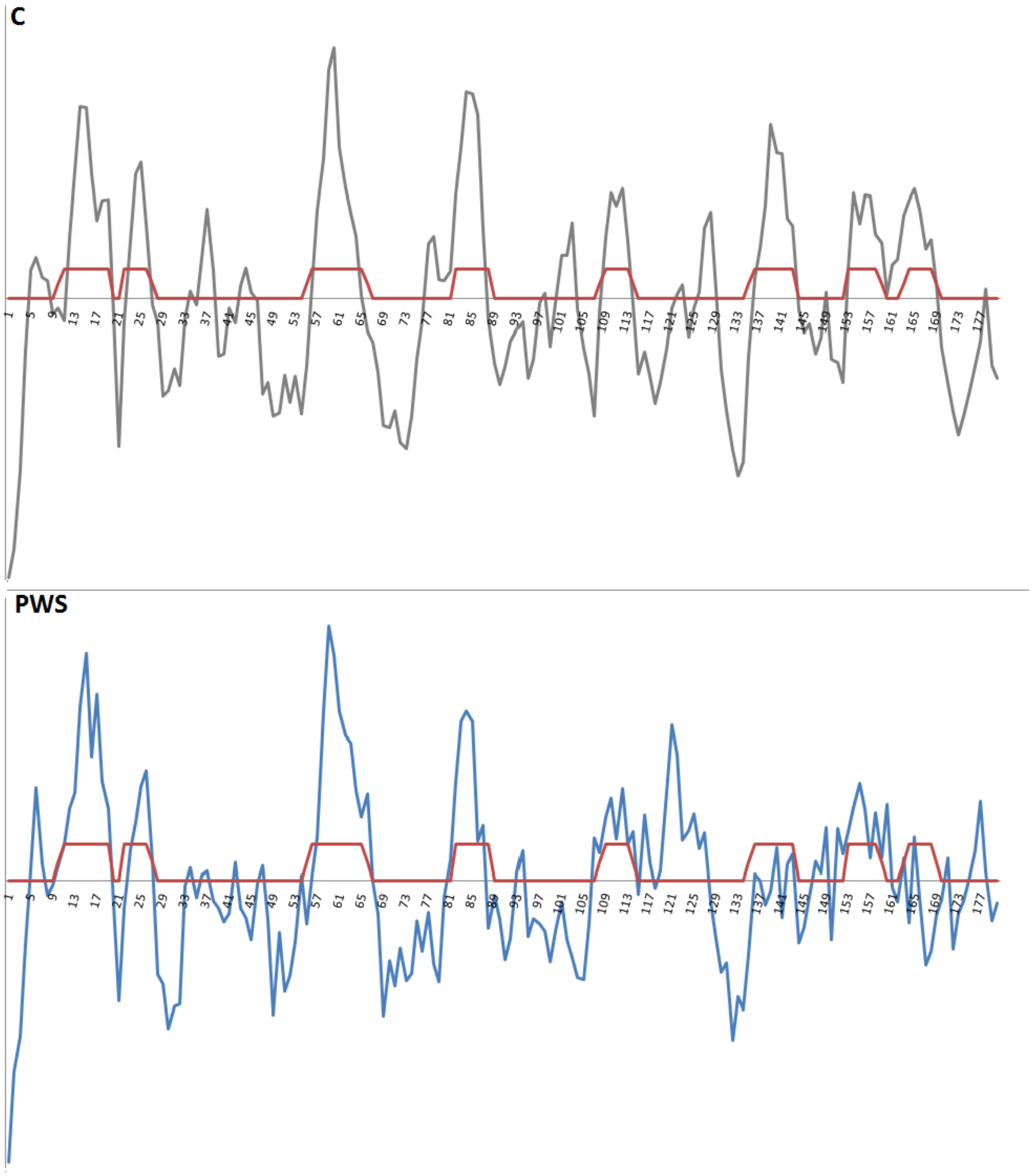

Supplementary Figure 1. Experimental paradigm. Red lines represent the baseline/activation regressor denoting disgust scene timing. The regressor was adjusted using the actual dynamic information obtained in an independent sample undergoing identical assessment. Grey line (top) and blue line (bottom) illustrate the time course of the fMRI signal (averaged time series from all subjects in the group) in a preselected region-of-interest showing robust disgust-induced activation, the left fusiform gyrus, of controls (C) and Prader Willi syndrome (PWS) patients, respectively. The numbers correspond to the scans.

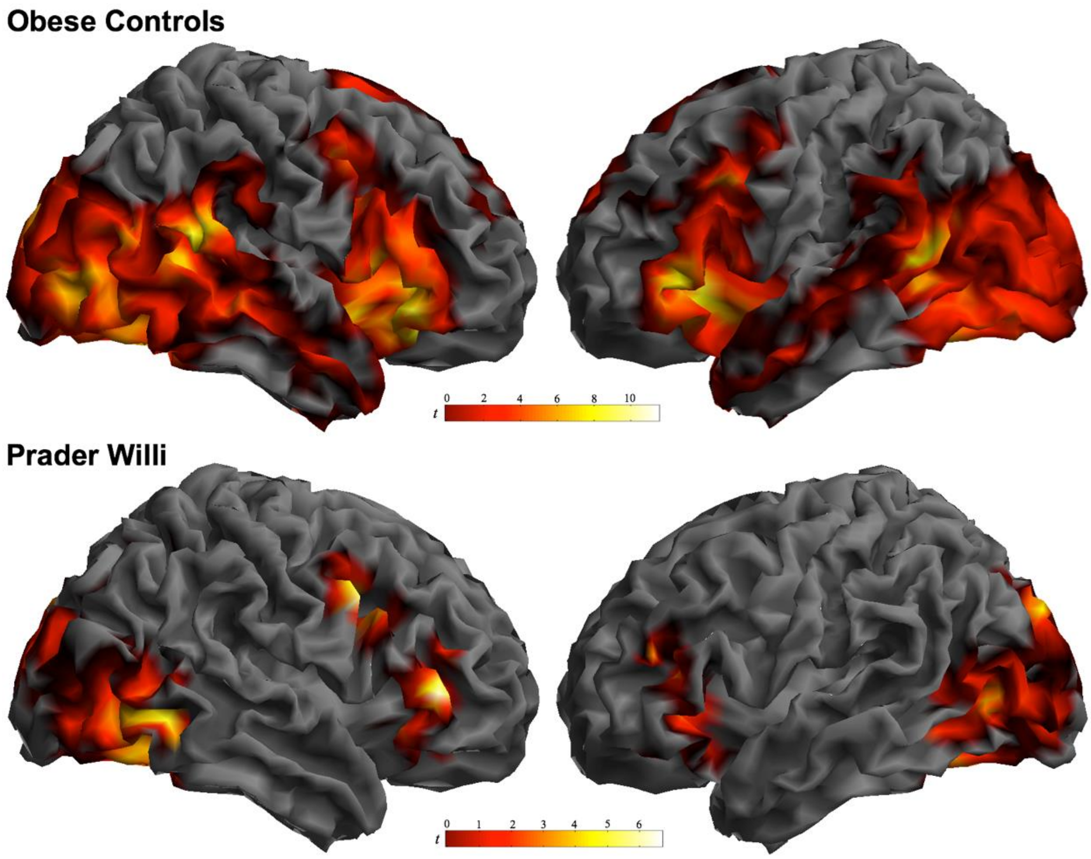

Supplementary Figure 2. Global brain response to disgusting food scenes compared with scenes of appetizing food.

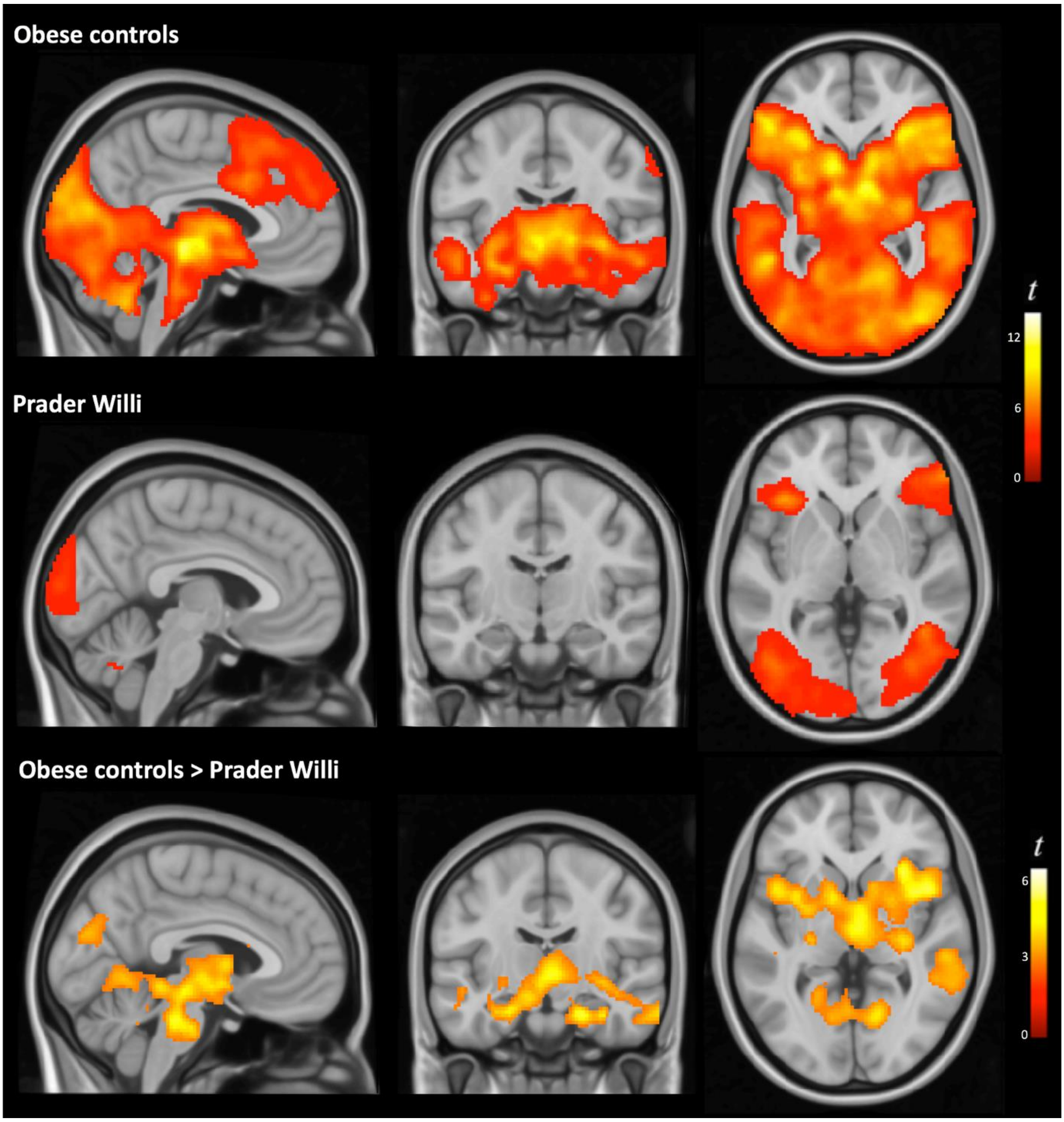

Supplementary Figure 3. Between-group differences in the brain response to disgusting food representations. Compared with BMI-matched control subjects, individuals with Prader Willi syndrome showed significantly decreased activation in several subcortical structures. Right side of the figure corresponds to the right hemisphere for both coronal and axial views.

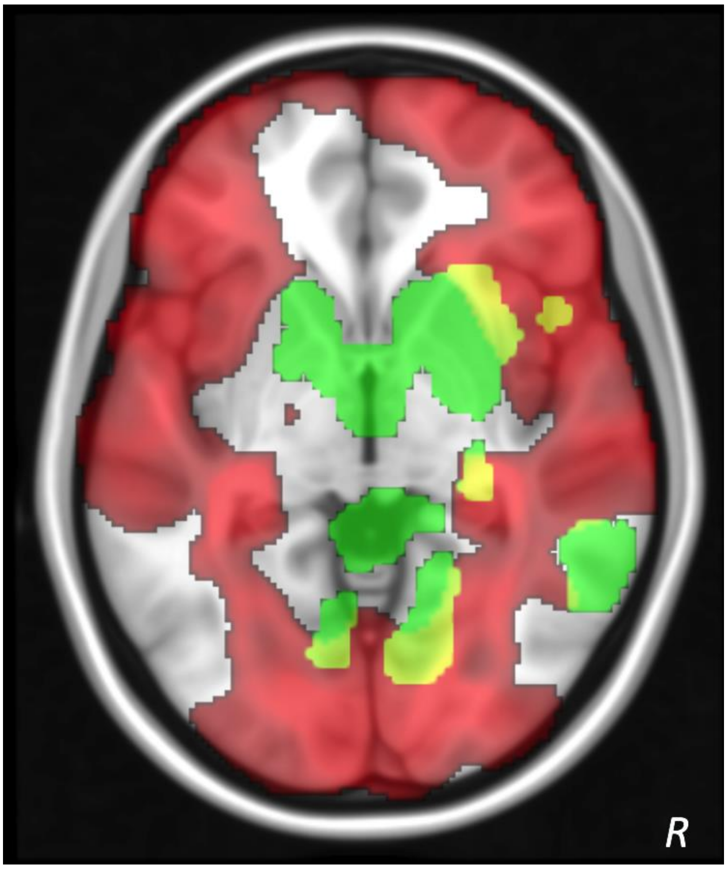

Supplementary Figure 4. Combined representation of brain regions showing decreased activation in response to disgusting food stimuli (green) and brain regions showing significant gray matter volume reductions (red) in patients with PWS. Gray matter data analysis was performed following the procedure fully described in Pujol et al. (Neuroimage. 2016 Apr 1;129:175-184). Note the limited overlap between both maps (yellow).

Supplementary Table 1

Table S1

Differences in brain activation between PWS patients and normal-weight controls

	**Controls > Prader Willi**	
**Region**	*x y z*	*t*
Left Hippocampus	-20 -20 -18	5.3
Right Hippocampus	28 -8 -24	4.9
Right Amygdala	28 -6 -22	4.6
Left Amygdala	-22 -8 -22	4.0
Right Basal Ganglia	22 0 -4	4.6
Left Basal Ganglia	-14 0 8	4.0
Thalamus	2 -10 4	4.4
Hypothalamus	-4 -4 -8	4.2
Periaqueductal Gray	4 -30 -12	3.9
Right Superior Temporal/Supramarginal gyrus	64 -38 32	4.9
Right Lingual gyrus	18 -72 4	4.4

PWS, Prader Willi syndrome. x y z, coordinates given in Montreal Neurological Institute (MNI) space. Statistics correspond to a threshold of $p<0.05$ Family Wise Error (FWE) whole-brain corrected at the cluster level using the Statistical nonParametric Mapping (SnPM13) software. The supra-threshold cluster size was set at 1,365 voxels. The total number of significant voxels was 14,662 (117.3ml).

Supplementary Table 2

Table S2

Differences in brain activation between PWS patients and BMI-matched controls

	**Controls > Prader Willi**	
**Region**	*x y z*	*t*
Left Hippocampus	-26 -10 -22	4.4
Right Hippocampus	28 -4 -22	5.1
Right Amygdala	32 -2 -14	5.5
Left Amygdala	-24 -6 -14	4.9
Right Basal Ganglia	22 4 -12	5.2
Left Basal Ganglia	-18 2 -8	6.3
Thalamus	4 -10 -2	4.6
Hypothalamus	-4 -8 -8	4.0
Periaqueductal Gray	4 -32 -12	3.9
Right Insula	46 10 4	5.7
Right Temporal cortex	54 -30 -6	4.5
Right Lingual gyrus	14 -66 4	4.3

PWS, Prader Willi syndrome. x y z, coordinates given in Montreal Neurological Institute (MNI) space. Statistics correspond to a threshold of $p<0.05$ Family Wise Error (FWE) whole-brain corrected at the cluster level using the Statistical nonParametric Mapping (SnPM13) software. The supra-threshold cluster size was set at 1,392 voxels. The total number of significant voxels was 19,831 (158.6ml).

Supplementary Table 3

Table S3

Significant peak activations observed during the disgust-evocation cycle in PWS patients

**Region**	Peak t-value	x y z	Scan of peak activation
Right Anterior Insula/Operculum	5.0	42 32 10	5
Left Anterior Insula/Operculum	3.1	-38 24 -2	5
Right Lateral Frontal cortex	5.0	50 6 32	11
Left Fusiform Gyrus	4.0	-34 -66 -12	8
Left Lateral Occipital cortex	5.0	-40 -78 8	9
Right Lateral Occipital/Fusiform Gyrus	3.5	46 -52 -2	9
Cuneus	3.7	0 -74 10	9

PWS, Prader Willi syndrome. x y z, coordinates given in Montreal Neurological Institute (MNI) space. Statistics correspond to a threshold of $p<0.05$ Family Wise Error (FWE) whole-brain corrected at the cluster level using the Statistical nonParametric Mapping (SnPM13) software. Scan #1 corresponds to the first scan after disgusting scenes onset.
